# Abilities and Disabilities—Applying Machine Learning to Disentangle the Role of Intelligence in Diagnosing Autism Spectrum Disorders

**DOI:** 10.3389/fpsyt.2022.826043

**Published:** 2022-03-03

**Authors:** Nicole Wolff, Matthias Eberlein, Sanna Stroth, Luise Poustka, Stefan Roepke, Inge Kamp-Becker, Veit Roessner

**Affiliations:** ^1^Department of Child and Adolescent Psychiatry and Psychotherapy, Faculty of Medicine, Technische Universität Dresden, Dresden, Germany; ^2^Institute of Circuits and Systems, Faculty of Electrical and Computer Engineering, Technische Universität Dresden, Dresden, Germany; ^3^Department of Child and Adolescent Psychiatry, Psychosomatics and Psychotherapy, Philipps University, Marburg, Germany; ^4^Department of Child and Adolescent Psychiatry and Psychotherapy, University Medical Center Göttingen, Göttingen, Germany; ^5^Department of Psychiatry, Campus Benjamin Franklin, Charité - Universitätsmedizin Berlin, Berlin, Germany

**Keywords:** autism spectrum disorders, IQ, intellectual disability, ADOS, machine learning, diagnostic, intelligence

## Abstract

**Objective:**

Although autism spectrum disorder (ASD) is a relatively common, well-known but heterogeneous neuropsychiatric disorder, specific knowledge about characteristics of this heterogeneity is scarce. There is consensus that IQ contributes to this heterogeneity as well as complicates diagnostics and treatment planning. In this study, we assessed the accuracy of the Autism Diagnostic Observation Schedule (ADOS/2) in the whole and IQ-defined subsamples, and analyzed if the ADOS/2 accuracy may be increased by the application of machine learning (ML) algorithms that processed additional information including the IQ level.

**Methods:**

The study included 1,084 individuals: 440 individuals with ASD (with a mean IQ level of 3.3 ± 1.5) and 644 individuals without ASD (with a mean IQ level of 3.2 ± 1.2). We applied and analyzed Random Forest (RF) and Decision Tree (DT) to the ADOS/2 data, compared their accuracy to ADOS/2 cutoff algorithms, and examined most relevant items to distinguish between ASD and Non-ASD. In sum, we included 49 individual features, independently of the applied ADOS module.

**Results:**

In DT analyses, we observed that for the decision ASD/Non-ASD, solely one to four items are sufficient to differentiate between groups with high accuracy. In addition, in sub-cohorts of individuals with (a) below (IQ level ≥4)/ID and (b) above average intelligence (IQ level ≤ 2), the ADOS/2 cutoff showed reduced accuracy. This reduced accuracy results in (a) a three times higher risk of false-positive diagnoses or (b) a 1.7 higher risk for false-negative diagnoses; both errors could be significantly decreased by the application of the alternative ML algorithms.

**Conclusions:**

Using ML algorithms showed that a small set of ADOS/2 items could help clinicians to more accurately detect ASD in clinical practice across all IQ levels and to increase diagnostic accuracy especially in individuals with below and above average IQ level.

## Introduction

Public awareness about autism spectrum disorder (ASD) is steadily increasing (1, 2). This is also reflected by the growing number of children, adolescents, and adults who use diagnostic services in outpatient clinics for ASD. Although, in the eyes of the public, the term autism is often associated with special talents and abilities (3), creating a somewhat distorted picture of the disorder, the larger part of the group of autistic people shows intellectual abilities below the average of the general population. Fombone (4) reviewed 20 epidemiological studies of ASD published from 1966 to 2001 and summarized that the median percentage of individuals with ASD and cognitive impairment was 70% (range 40–100%). More recent epidemiological studies reported that about 56% of people with ASD have an IQ <70 (5) or 31% of children with ASD are classified in the range of an intellectual disability (ID), 25% in the borderline range (IQ 71–85), and 44% have IQ scores in the average to above average range (i.e., IQ > 85) (6). This heterogeneity of data about IQ in ASD is in line with statements that IQ might be *the* source of heterogeneity of ASD as a heterogeneous (group of) disorder(s) (7–9).

The IQ is associated with the individual level of functioning and—particularly in ASD—with the ability to acquire and apply specific skills in order to handle typical everyday situations, to plan and act with foresight and to be flexible and adaptive to changing environmental conditions (9, 10). Knowledge regarding the individual IQ is thus important to gain more knowledge about the functionality of the person with ASD. This, in turn, is relevant for both diagnostic accuracy and the treatment planning and success.

It has been observed that ASD symptom measures such as the Autism Diagnostic Observation Schedule (ADOS), the Autism Diagnostic Interview Revised (ADI-R), and Social Responsiveness Scale (SRS) capture much more than just the pure symptoms of ASD: increased scores may reflect rather additional impairments than ASD-related social communicative deficits and repetitive behaviors alone (11). IQ-related particularities increase the risk for drawing incorrect conclusions about etiological and phenotypic relationships (11). Such incorrect conclusions may result in false-negative or false-positive decisions in ASD diagnosis.

Within the so-called gold standard clinical diagnostics for ASD, a standardized interview conducted with the caregiver of the individual (ADI-R), a standardized behavioral observation of the individual itself (ADOS/2), a differential diagnostic examination, and an IQ testing are included. Based on all this information, individuals are categorized according to the psychiatric multiaxial schema (12, 13). Within this schema, individuals will be categorized in six axes: (1) psychiatric syndromes; (2) circumscribed developmental delay; (3) intellectual level or mental retardation; (4) somatic symptomatology; (5) associated current abnormal psychosocial circumstances; and (6) global assessment of psychosocial stressors and adaptive functioning. With respect to axis 3, the intellectual level, a rough categorization based on the tested or estimated IQ is made: level 1: IQ > 129, level 2: IQ = 115–129, level 3: IQ = 85–114, level 4: IQ = 70–84, level 5: IQ = 50–69, level 6: IQ = 25–49, level 7: IQ = 20–34, and level 8: IQ <20 (13).

Although the ADOS/2 offers good sensitivity and specificity values (14, 15), there are frequent cases in which the diagnosis is either missed (false negative) or given incorrectly (false positive)—false particularly in the retrospective consideration of changes in phenomenology and thus diagnoses during development over several years (16). There are several potential reasons facilitating false-positive or false-negative cases, like under-resourced familial or educational environment, presence of only subtle ASD symptoms, and presence or absence of coexisting or differential diagnoses [e.g., anxiety disorder (17), ADHD (18), or psychosis (19)]. In addition, high intelligence enabling a partial compensation of ASD-related social and communicative difficulties may be an influencing factor (20). Although individuals with ASD may benefit from high intelligence by potentially higher compensation abilities, it was also observed that individuals with ASD with above average IQ perform low in domains of facial and emotional identification, visual pattern recognition, and verbal working memory (7). Thus, individuals with ASD with above average IQ also show impairments, which may be targeted in clinical assessment and treatment. Individuals with low intelligence are also susceptible to ASD misidentification—either because an intellectual disability (ID) diagnosis overshadows ASD symptoms or because cognitive impairments are falsely interpreted as ASD (21–23). As the accuracy of psychiatric diagnoses affects both the preferred therapy choice and the respective outcome (24), and implications from obtaining a lifelong diagnosis such as ASD can be severe, the diagnostic accuracy should be as high as possible. Therefore, clinicians should be aware of specific individual characteristics, which are concomitant with different levels of IQ and which may lead to increased or decreased ADOS/2 scores (16).

The present study aimed to (1) analyze the accuracy of the ADOS/2 algorithm by comparing the reaching and exceeding of the ADOS/2 cutoffs with the BEC ASD diagnosis also for sub-cohorts defined by different IQ levels. (2) In addition, we investigated whether these accuracies can be increased by applying data-driven machine learning (ML) approaches that processed additional features including the IQ level. Finally, (3) with the help of ML approaches, we aimed to identify which of the considered features are most important to discriminate accurately between participants with and without a BEC ASD diagnosis to focus clinicians' attention on features that best distinguish between ASD and Non-ASD, so that the most accurate, economical, and comprehensive classification of each individual is achieved.

## Materials and Methods

### Participants

Data from participants originate from a large German research consortium called ASD-Net (25) and were obtained from four specialized centers in Germany. All included participants of our data received the BEC diagnosis or did follow the ASD diagnostic gold standard procedure that was applied by specialized clinicians in all centers. Data from more than 2,500 patients were collected retrospectively from medical records (retrospective chart review) and analyzed anonymously, with approval from the local ethics committee (Az. 92/20). Due to the retrospective nature of data collection and analysis based on anonymized data, there was no need for further informed consent. All methods were applied in accordance with relevant institutional and international research guidelines and regulations. From the ASD-Net database, all participants with available information about their IQ level and ADOS data were selected (*N* = 1,084; 40.6% with ASD, 5.5% with ID). Data of the applied IQ tests were not available for all included individuals; for some individuals, IQ levels were only present in terms of clinical impressions. IQ level 8 was not assigned within the sample. Sample characteristics are given in Table 1.

**Table 1:** Sample characteristics.

	**Non-ASD**		**ASD**		**Non-ID**		**ID**	
	***N***	**M (SD)**	***N***	**M (SD)**	***N***	**M (SD)**	***N***	**M (SD)**
Age	644	13.4 (±9.7)	440	14.9 (±11.1)	1,024	14.1 (±10.4)	60	12.9 (±8.9)
IQ	545	99.3 (±21.1)	370	101.2 (±26.0)	892	101.2 (±22.4)	23	57.3 (±9.6)
IQ level	644	3.2 (±1.2)	440	3.3 (±1.5)	1,024	3.1 (±1.2)	60	5.5 (±0.7)

*Total number of participants is N = 1,084. Patients were chosen based on the presence of information about their IQ level*.

### Measures

Information regarding the IQ level of the individuals was recorded with respect to the psychiatric multiaxial schema, in which axis 3 describes the level of intelligence. Hence, intelligence can be either measured psychometrically or assessed clinically. Please note that although individuals from IQ level 5 to 8, i.e., IQ ≤ 70, fulfill the diagnostic criteria of ID according to the psychiatric multiaxial system, the diagnosis ID was assigned to only 28.9% of the cases in clinical practice. Therefore, presence or absence of an ID was included in all analyses as a separate factor in addition to IQ level. The four modules (the toddler module has not been included) of the ADOS (26) consist of 29, 27, 28, or 31 items, respectively. The ADOS items (hereafter abbreviated to “items”) are rated by a trained psychologist from zero (inconspicuous) to three (conspicuous) or, in a few cases, with seven or eight (indicating a conspicuous behavior, which is not to be evaluated in the sense of autism or indicating the impossibility to rate this item, e.g., because the individual shows too few vocalizations for intonation to be judged). Individuals with more than four missing items in their respective module's protocol were discarded from analyses. The final BEC diagnosis was coded as 0 for Non-ASD and 1 for ASD and will be the dependent variable in all following analyses.

### ADOS/2 Algorithm

To apply the ADOS/2 algorithm, the item-rating three is converted to two; similarly, seven and eight are converted to zero. Depending on the applied module, 11 to 16 items are summed up for each individual participant. If module-specific cutoffs are exceeded, the diagnosis of ASD is suggested by the ADOS/2 algorithm. However, according to the ASD diagnostic gold standard procedure, the BEC diagnosis that is given by the trained expert is also based on the clinical impression and can differ from the result of a very well-established diagnostic instrument as the ADOS/2. The ADOS/2 algorithm's accuracy is obtained by comparing the ADOS/2 cutoff with the BEC ASD diagnosis.

### Data-Driven Machine Learning Approaches

In contrast to the ADOS/2 algorithm, the estimated ML algorithms are not module-specific. Therefore, the recoded data from all ADOS modules have been combined to one data set before being processed by the ML algorithms. The intention was to increase robustness of the model estimation by increasing the number of samples available for training a single model. Hence, a single model is trained on the whole data instead of training four module-specific models on the modules' respective data only.

In order to be able to process all individuals congruently, similar labeled (and thus assumingly corresponding) items from different modules have been identified and were arranged accordingly; missing items were filled with−5. This value was chosen, as it is clearly out of range, allowing the algorithms to use the information whether data were collected or not. In the present paper, the naming of the items used is listed in module order, i.e., module 1/module 2/module 3/module 4. Furthermore, the naming refers to the ADOS convention: indication of initial letters (A–D) for the respective dimension plus a number (indicating the temporal sequence of the item). There are items that occur in all four modules (*N* = 17), items that occur in three modules (modules 2–4, *N* = 3), and items that occur in two modules (modules 1 and 2, *N* = 6; modules 3 and 4, *N* = 7). If items do not appear in all modules, they are marked with a “-” in the appropriate place to indicate that they do not appear in that module.

To offer two examples: (a) item “B3/B2/B2/B2” indicates the item “socially directed facial expression,” which is item B3 in module 1 and item B2 in modules 2 to 4. In addition, it is also visible that this item is included in all four modules. In contrast, (b) item “-/-/B6/B7” indicates the item “social insight,” which is part of modules 3 (B6) and 4 (B7). In modules 1 and 2, however, this item does not occur and is therefore coded as−5 for all participants of these modules.

Besides the items, all potentially relevant additional information that is collected during standard diagnostic procedure was provided to the ML algorithms, i.e., the applied ADOS module, IQ level, age of the individual, sex of the individual, and presence or absence of an ID. In total, the number of features processed by the ML algorithms summed up to 49 for each subject, independently of the applied ADOS module. All individual features can be identified in **Figure 4** in the Results section.

These features were processed by decision trees (DT) with the aim to discriminate subjects with a BEC ASD diagnosis from subjects without as accurate as possible. DTs are simple models that are inherently interpretable due to their flowchart-like structure where nodes are representing categorical tests that are applied to individual features.

DTs are grown node by node, always aiming to find a condition and a factor that split the data in homogeneous groups of individuals with ASD and individuals without ASD. The inhomogeneity (or impurity) of a group can be measured, e.g., with the entropy or the gini-index of the depended variable, i.e., the diagnosis variable. The reduction of the impurity measure (or impurity decrease) is used during training for choosing appropriate features and conditions for each node (27). Here, the reduction of the impurity measure is defined as the difference of the impurity before the split and the average impurity of the two samples after the split, weighted with their respective number of samples in the training data.

While DTs are natively interpretable, they can be sensitive to minor changes in input data and often suffer from suboptimal classification performance (27). To overcome these limitations, outputs from multiple DTs can be combined, forming an RF. Usually, each tree is trained on randomly sampled subsets of the original data. While RFs are more powerful and typically superior in terms of stability and accuracy, their complexity comes at the cost of reduced interpretability of their decision process.

#### ML Stage 1: Parameter Selection

The available data were randomly split in 80% training and 20% test data. The relative frequencies of IQ level were preserved in both data sets. In order to determine optimal (in terms of predicting the BEC diagnosis) model parameters for the DT, a fourfold stratified cross-validated grid search was performed on the training data. For this procedure, all possible combinations of predefined parameter sets are compared. The best parameters were chosen based on the cross-validated out-of-bag accuracy on the training data only. Afterwards, multiple trees with optimized parameters were combined to an RF. Each tree in the ensemble was trained on a different set of samples, drawn randomly with replacement from the training set. This procedure is called bootstrap. The optimal number of estimators used for the RF as well as the number of randomly drawn samples for each bootstrap is again determined in a fourfold stratified cross-validated grid search on the training data.

#### ML Stage 2: Performance Estimate on Unseen Data

In order to estimate the performance of the optimally parameterized models on unseen data, they were subsequently trained on the whole training data and tested on the 20% held-out test set.

#### ML Stage 3: Performance Estimate on Unseen Data

To obtain out-of-sample classifications for the whole available data set, a fivefold stratified cross-validation was used. The classifiers were trained on four splits and evaluated on the fifth. Subsequently, the predictions of the five test splits were combined.

##### Identification of Best Discriminating Features

Finally, for the identification of discriminating features by the aid of ML algorithms (aim 2), the algorithms were parameterized according to the previously determined optimal parameters (aim 1, Stage 1). In order to allow all available samples to contribute to this analysis, the whole data set was used for training. Subsequently, based on the trained models, the relevance of the individual features in regard of the discrimination between participants with and without finally diagnosed ASD according to ASD gold standard procedure was analyzed.

For the DT, the decision paths are visualized and discussed. Together with the distribution of the data, the discriminating power of individual features can be determined intuitively in this structure.

An RF constitutes of up to hundreds of DTs, which all contribute to the final decision. Therefore, an explicit description of the decision rules of a RF would be too complex to obtain an intuition about relevance of individual features. Hence, the importance of features for the trained RF will be considered only statistically.

There are numerous methods for estimating importance of input variables for RF or ML algorithms in general (28). Here, the reduction of the impurity measure is used, averaged over all training samples as well as all nodes of the RF that are associated with the respective factor.

Data analysis and visualization were implemented in python, using the packages scikit-learn (29), pandas (30), matplotlib (31), and scipy (32). The used code is available under URL (https://github.com/MatthiasEb/ASD-IQ). As the original data that were used for the presented results cannot be published as they contain clinical information, sample data are provided.

## Results

### IQ Level Distribution

**Figure 1:**
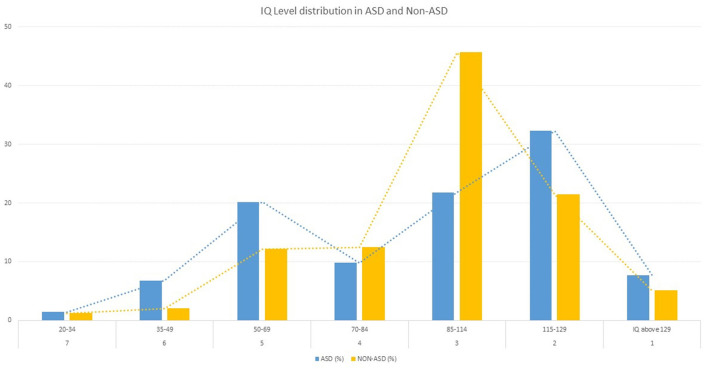
IQ level distribution (from IQ level 7-1 in %) of ASD (n = 440) and non-ASD (n = 644) group in a German sample referred to specialized ASD clinics.

Of the 440 individuals with ASD, 38.2% had a below average IQ level (IQ level ≥ 4, IQ <70), while 8.2% had moderate to severe intellectual disability (IQ level ≥ 6, IQ <50); 21.8% had average intelligence (IQ level = 3, 114 > IQ > 85) while 40% had an above average IQ level (IQ level ≤ 2, IQ > 115) (see Figure 1). In contrast to the 644 individuals without ASD, 27.7% had a below average IQ level (IQ level ≥ 4, IQ <70), while 3.2% had moderate to severe intellectual disability (IQ level ≥ 6, IQ <50); 45.7% had average intelligence (IQ level = 3, 114 > IQ > 85) while 26.5% have an above average IQ level (IQ level ≤ 2, IQ > 115). Differences between individuals with ASD and Non-ASD are significant χ^2^ = 85.93, *df* = 6, *p* < 0.001.

Although 38.2% of the individuals with ASD had a below average IQ level (IQ level ≥ 4, IQ <70) and therefore fulfill the diagnostic criteria of ID according to the psychiatric multiaxial system, the diagnosis ID was given only in 28.9% of the cases.

### ADOS/2 Algorithm Accuracy (Aim 1) and Comparison to ML Approaches (Aim 2)

**Figure 2:**
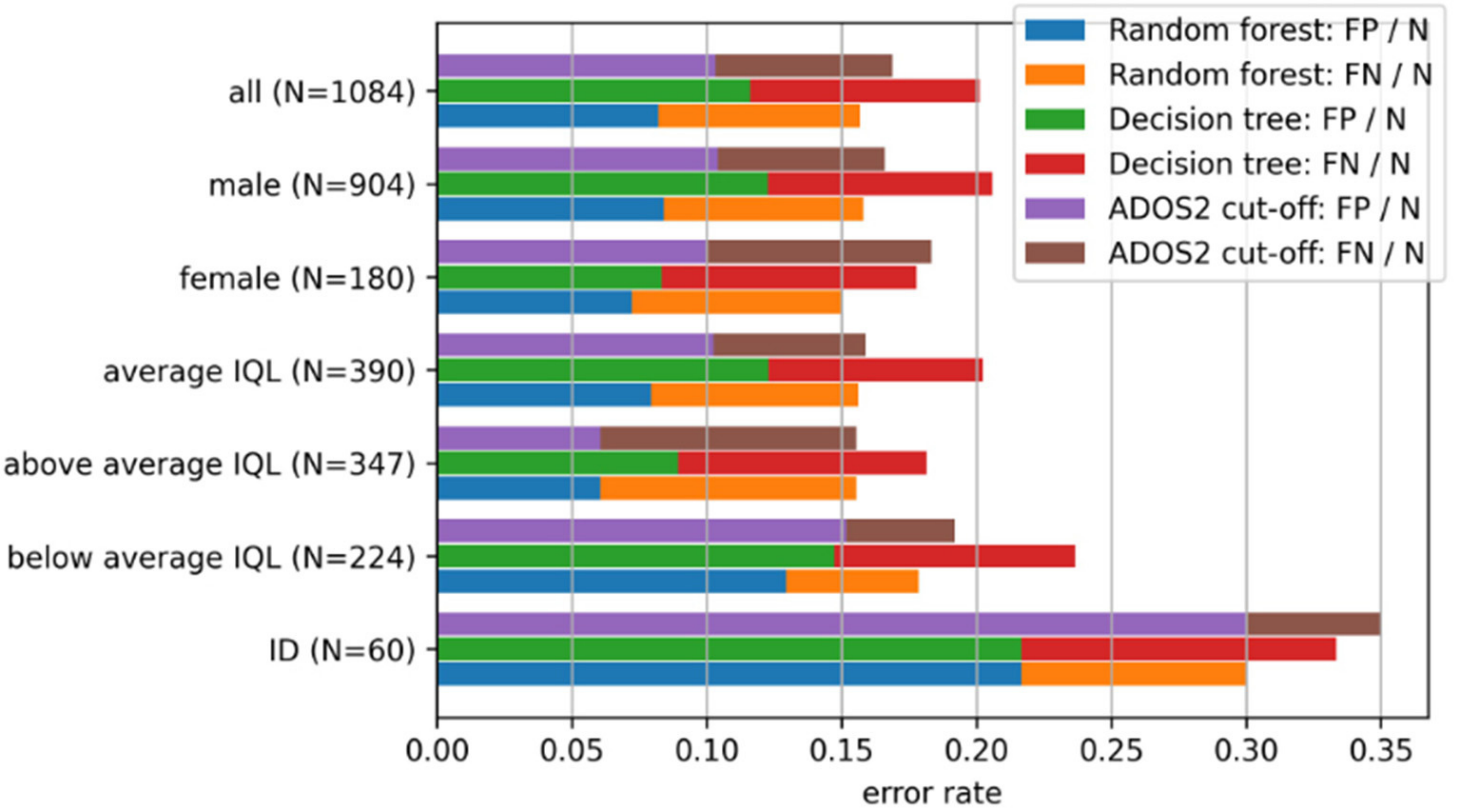
Comparison of error rates and their composition of false negatives (FN) and false positives (FP) by several sub-cohorts (e.g. sex-specific). For the calculation of the error rates of the respective algorithm [random forest (RF), decision tree (DT), ADOS/2] per sub-cohort, FN and FP are divided through the respective sample size N of the sub-cohort. IQL, IQ level.

For all participants, the cutoffs according to the ADOS/2 algorithm match the final BEC ASD diagnosis in 83.87% across all IQ levels. The sub-cohort-specific error rates and their respective composition of false positives and false negatives are shown in Figure 2.

The results of the individual stages of the application of DTs and RFs described in the previous section are given in the following:

#### ML Stage 1

The investigated parameters, their respectively considered values, as well as the optimal choices are given in Table 2. The mean accuracy to predict the final BEC ASD diagnosis of the four cross-validation runs with optimally parameterized DTs was 80.74%. Analogously for the RF, the highest observed cross-validated accuracy for all grid-search runs was 84.08%.

**Table 2:** Parameters considered for grid search.

	**Parameter**	**Values**
Decision Tree	Maximal depth	2, 3, 4, 5^*^, 7, 10, 15
	Complexity parameter for Minimal-Complexity pruning	0^*^, .1, .5, 1 5, 10
	Splitting criterion	Gini index, Entropy^*^
	Minimum number of samples for split	2^*^, 3, 4, 5, 10
	Minimum number of samples per leaf	1, 2, 4, 8^*^, 16
Random Forest
	Number of estimators	2, 4, 8, 16^*^, 32, 64, 128, 256
	Number of samples drawn for the training of the individual trees, in proportion to the number of samples in the train set	0.3, 0.5, 0.7, 0.8, 1^*^

* *Parameters that were found to be optimal to predict the final BEC ASD diagnosis*.

#### ML Stage 2

In order to estimate the accuracy of the optimally parameterized models on unseen data, both were subsequently trained on the whole training data and tested on the held-out test set. A test accuracy of 83.87 and 86.16% was achieved for the DT and the RF, respectively.

#### ML Stage 3

On the whole data set, the accuracies to predict the final BEC ASD diagnosis are 84.08% for a single DT classifier and 86.16% for the RF, respectively. The sub-cohort-specific error rates and their respective composition of false positives (FP) and false negatives (FN) as well as the comparison to the ADOS/2 algorithm performances are shown in Figure 2. Differences in FPs and FNs of the ADOS/2 algorithm between the listed cohorts are tested by paired *t*-tests. For the ADOS/2 algorithm, the relative number of FPs of patients with ID is increased by a factor of approximately three compared to patients with average IQ level (*T* = −33.13, *df* = 199, *p* < 0.001). Even for individuals without a diagnosis of ID but with a below average IQ level (IQ ≤ 84), the number of FPs is increased by a factor of 1.5 (*T* = −12.88, *df* = 199, *p* < 0.001). For individuals with an above average IQ level (IQ ≥ 115), the relative number of FNs is increased by a factor of 1.7 compared to patients with an average IQ level (*T* = −21.84, *df* = 199, *p* < 0.001).

In order to estimate the confidence of an increase in performance between RF and ADOS/2 cutoff, 200 bootstraps with *N* = 900 were sampled from the original data. Sampling was performed with replacement. Out-of-sample predictions of the RF as well as for the ADOS/2 cutoff were calculated on the remaining data. Subsequently, the performances of the RF classifiers and the ADOS/2 cutoffs were compared for these 200 sets. An average increase in accuracy of 1.86 ± 1.17% was observed for the RF, the probability of the RF performing equally or worse than the cutoff could be estimated to 8%.

### Identification of Discriminating Features (Aim 3)

**Figure 3:**
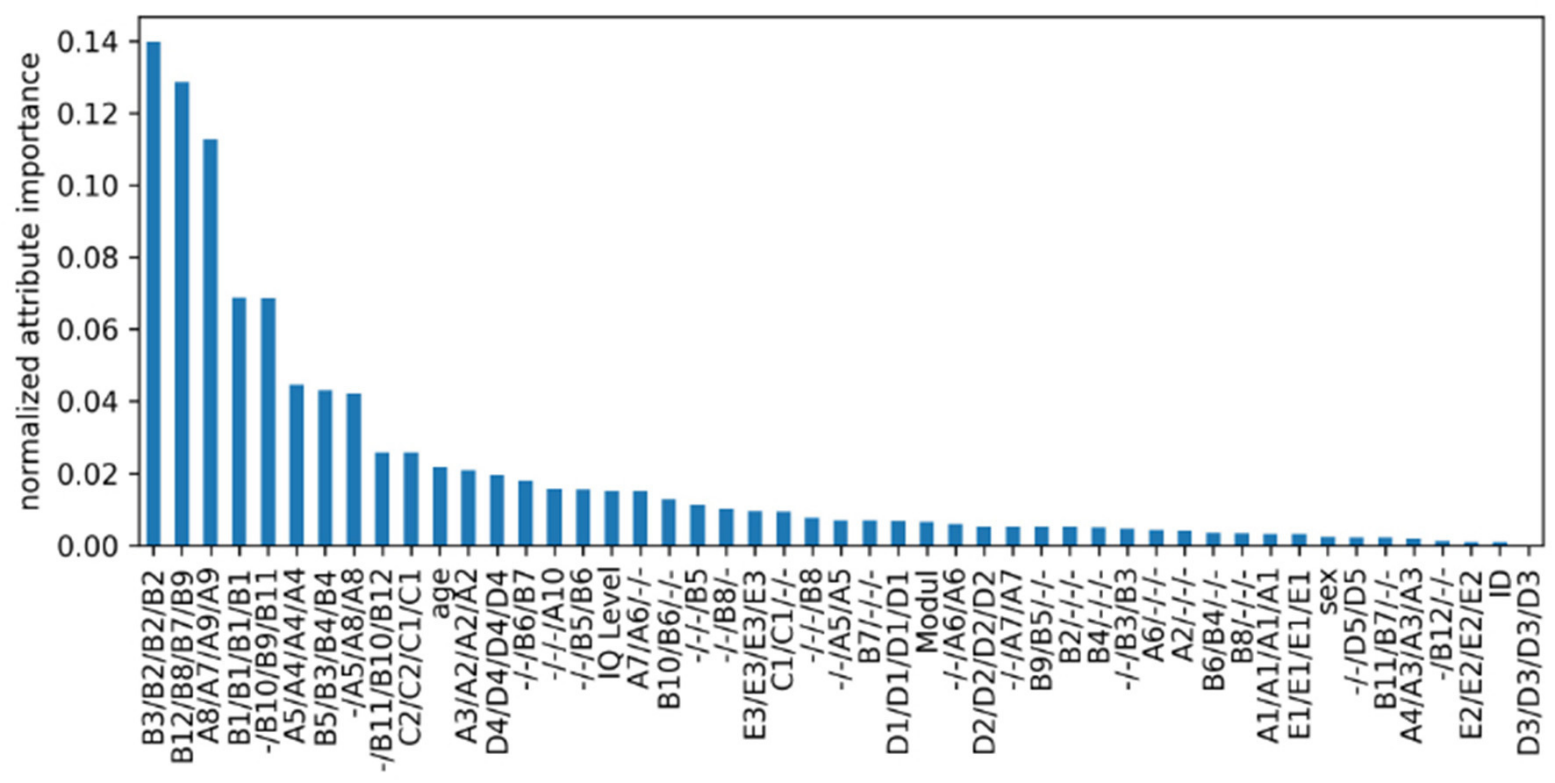
Decision tree with optimal parameters, trained on the full dataset. Its in-sample accuracy (i.e. on the training data/the full dataset) is 84.08%. Samples are passing the tree from top to bottom. If the conditional test associated with a node is passed, the left child-node will be visited, else the right node. Furthermore, the number of samples, proportion of ASD samples in the training set are given as well as the estimated class. The color codes the “purity” of the sample: From blue (100 % ASD) over white (50% ASD) to orange (0% ASD). For the ADOS-items, the names of corresponding items in all four different modules are given, separated by “/”. If no corresponding item for a given module could be found, they are marked as “-”.

One DT parameterized optimally to predict the final BEC ASD diagnosis (according to aim 1, stage 1), trained on all available data, is shown in Figure 3.

We observed that solely the item B3/B2/B2/B2 (“Socially directed facial expression”) could be sufficient to suggest that the participant has presumably no ASD. In more detail, if B3/B2/B2/B2 is rated as inconspicuous (value 0), the likelihood to diagnose ASD is only 11.5% (see Figure 3). If additionally the item “quality of social responses” (-/B10/B9/B11) is rated as inconspicuous (value 0) or no data were collected (value−5), the likelihood to diagnose ASD is only 4.7% (*N* = 344). As -/B10/B9/B11 is not part of module 1, it is therefore−5 for all participants from this module. As a consequence, the DT classifies all participants from module 1 with inconspicuous rating for B3/B2/B2/B2 as Non-ASD (*N* = 22, 9% received ASD diagnosis).

In contrast, if B3/B2/B2/B2 is conspicuous (value 1–3), the likelihood to diagnose ASD is 63% (37% receive no ASD diagnosis). If additionally the items B12/B8/B7/B9 (“quality of social approach”) and A5/A4/A4/A4 (“stereotypical/idiosyncratic language use”) are conspicuous (value 1–3) as well, 86.3% of the analyzed cases (*N* = 190) receive an ASD diagnosis. This suggests that, depending on the outcome of the before-mentioned items, very few (i.e., 1–3) items can be sufficient to obtain an accurate estimate of the likelihood of an ASD diagnosis.

**Figure 4:**
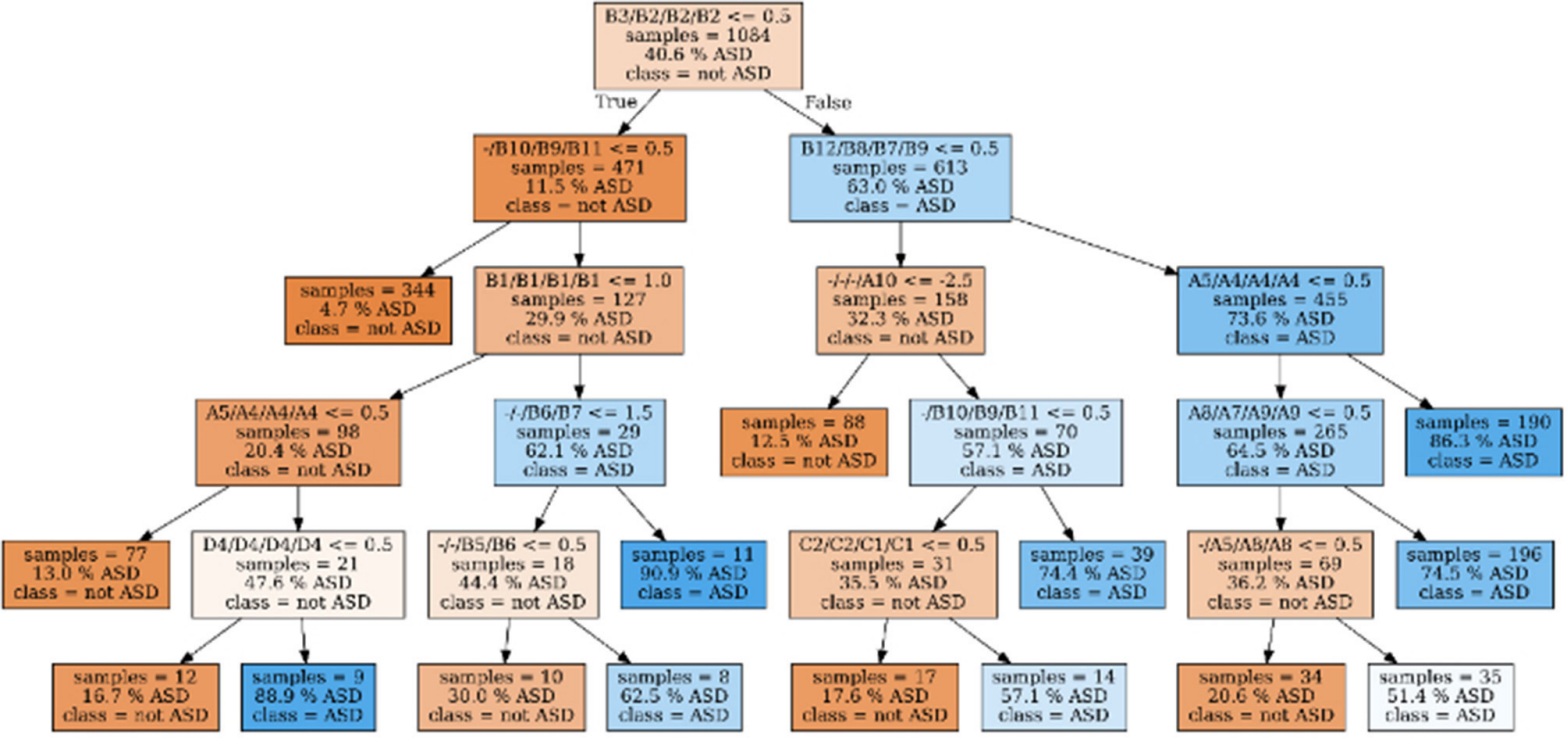
Feature importance for the trained random forest classifier. Features' importance have been normalized so that they sum to 1. All features used in this study are shown. The features corresponding to ADOS items are reported in the order M1/M2/M3/M4 according to their content. Features that do not appear in all modules are marked with a “-” to indicate that they do not appear in the respective module.

The mean reduction of the entropy of an RF parameterized as indicated in Table 2 and trained on all available data is shown in Figure 4. We observed that most features that are rated as relevant for the RF also appear in the DT: All of the six most descriptive features across the four modules (B3/B2/B2/B2, B12/B8/B7/B9, A8/A7/A9/A9, B1/B1/B1/B1, -/B10/B9/B11, and A5/A4/A4/A4) for the RF also appear in the presented DT. Additionally, as each of their position is relatively close to the root node, these items can be further considered highly relevant for the tree as well. Furthermore, it can be observed that the additional features provided to the algorithm contribute, if at all, only moderately to the decisions (i.e., age and IQ level, see Figure 4).

## Discussion

Within this study, we aimed to clarify whether the existing ADOS/2 algorithms are accurately discriminating between individuals with/without ASD with different IQ levels (aim 1). Furthermore, we compared the accuracy of ADOS/2 algorithms with ML algorithms that processed additional information, namely, the ADOS module, IQ level, age of the individual, sex of the individual, and presence or absence of an ID (aim 2). Finally, we examined discriminating features for distinguishing between ASD and Non-ASD based on these ML algorithms (aim 3).

To analyze the accuracy of the ADOS in different IQ levels, we initially looked at the IQ distribution of our sample and observed that solely 8.6% of the individuals with ASD have a co-existing diagnosis of ID, which is considerably low in comparison to former studies. For example, Baio et al. (6) reported 31% of children with ASD to be classified in the range of an ID. However, 28.4% of the individuals with ASD of our sample had an IQ <70, which is closer to the reported values of the epidemiological study by Baio et al. (6). In addition, we observed that 40% of our sample of individuals with ASD had an IQ level above average (IQ > 115), which is higher than in former studies (6, 33). For example, the latter reported that 3% of the children and adolescents with ASD had an above average IQ.

### ADOS/2 Algorithm Accuracy (Aim 1) and Comparison to ML Approaches (Aim 2)

Overall, we observed that the accuracies of 86.16% (RF), 84.08% (DT), and 83.87% (ADOS/2) are comparable across the ML (RT and DT) and the ADOS/2 algorithm (see also Figure 2). These accuracies are generally in line with recent studies from our groups and others (34, 35). Also, higher accuracies have been reported [e.g., Stroth et al. (36), who even observed an accuracy of 98.27% and 98.66% in ADOS Modules 2 and 3], probably due to module-specific analyses resulting in more homogeneity of the analyzed sample. Only a minor increase in total accuracy of 1.21% could be observed for RFs compared to the ADOS/2 cutoff.

### Sub-cohorts—Influence of IQ

All three algorithms (DT, RF, and ADOS/2 cutoff) are coherent in their performance in patient sub-cohorts with different IQ levels. Especially for individuals with an average IQ level (IQ: 85–114), no significant differences in performance could be observed. For individuals with below average IQ and ID, as compared to individuals with average IQ, false-positive diagnoses were significantly increased by the factor 2–3, when the ADOS/2 cutoff is applied. This confirms our hypothesis as well as the clinical impression (namely: the ADOS/2 algorithm is potentially overestimating ASD symptoms in sub-cohorts with very low cognitive abilities) and negatively influences the validity of the final BEC. This higher rate of false-positive diagnoses is alarming, given that 38% of our sample of individuals with ASD have a below average IQ. Previously, a false-positive diagnosis in individuals with a below average IQ has been suggested to be less devastating than a false-negative diagnosis of individuals with ASD (37). However, Kamp-Becker et al. were rather critical of this statement, arguing that a cutoff resulting in a false-positive ASD diagnosis in individuals with developmental delay is rather confusing in clinical practice, affecting both the individual with ASD and his social environment. As an alternative, the authors advocated the introduction of two cutoffs: a higher threshold for “higher specificity” and a lower threshold for “higher sensitivity” (38). Somewhat in line with the potential benefit of two ADOS/2 cutoffs, we observed that the false-positive rate in both individuals with below average IQ and with below average IQ plus ID can be reduced by applying alternatives to the ADOS/2 cutoff, as shown here with DTs or RFs. However, this increase in specificity comes at the cost of a lower sensitivity, i.e., an increase in the false-negative rate (which was not statistically significant).

In addition to the described high rate of false-positive diagnoses in individuals with below average IQ levels and with below average IQ plus ID, we detected a significantly increased number of false negatives (i.e., individuals with ASD, who did not reach ADOS/2 cutoff but have the BEC diagnosis ASD) for individuals with above average IQ levels (IQ ≥ 115) as compared to individuals with average IQ levels. This amount of false negative diagnoses is also concerning, given the finding that 40% of our ASD sample have an above average IQ level. However, the amount of individuals with an above average IQ level differs with respect to age and applied module. While, for example, within M4, 65.3% of individuals with ASD and a mean age of 26.07 years ± 12.28 have an above average IQ, in M3, this is true for 44.6% (mean age of 10.06 years ± 2.52). Within M2, solely 9.8% of individuals with ASD have an above average IQ (mean age of 8.15 years ± 4.03), and within M1, 1.5% (mean age of 6.33 years ± 3.27). Thus, especially for individuals who were diagnosed later in life (about 10 years and older), this observation may be explained by better adaptive performance during the diagnostic assessment and more efficient as well as compensatory strategies, like camouflaging, to mask or hide social difficulties (39–41). However, this is a *post-hoc* assumption and should be investigated in further studies.

Nevertheless, the increase in false positives for individuals with below average IQ levels as well as the increase in false negatives for individuals with above average IQ levels was independent of the algorithm applied in the present study but strongest in the ADOS/2 algorithm. As the BEC diagnosis of ASD is dependent not only on ADOS/2 results but also on the clinical impression as well as on further diagnostics, individuals with similar ADOS/2 results can receive different diagnoses. From this point of view, conspicuous behaviors during ADOS/2 assessment situations are seemingly not sufficiently specific for distinguishing between individuals with below average IQ plus ID and individuals with ASD, which is in line with recent research (42). However, further studies are needed to confirm this hypothesis.

#### Identification of Relevant Discriminating Features (Aim 3)

With respect to DT analyses, we observed that for the differentiation between BEC diagnosis of ASD yes vs. no, (a) very few items are relevant, and (b) for most of these items, it is sufficient to know that the behavior is inconspicuous [0]; further gradations (1–3) are not necessary.

The starting item within the DT is B3 “Socially directed facial expression,” which was observed to be sufficient to suggest that the individual has presumably no ASD. The relevance of this item within M1 was previously confirmed (34), but also somewhat contradicts findings from our group and another group (36, 43). Moreover, for M2–4, the likelihood to exclude ASD is even higher, by adding the Item “quality of social responses” (-/B10/B9/B11). The high relevance of that item was previously confirmed for all three Modules (2, 3, 5) by others (34, 44) and our group (35, 36).

These insights from data-driven ML models are consistent with the features that contribute to the calculation of the ADOS/2 cutoff: The four features with the highest average impurity decrease are relevant for the calculation of the ADOS/2 cutoff in all modules. Furthermore, all nine top-rated RF features contribute to the ADOS/2 cutoff in two or more modules.

Nevertheless, ADOS/2 cutoff relevant items differ between the four modules. Offering *post-hoc* suggestions to optimize the performance of module-specific cutoff calculation is thus difficult, since we could not analyze the data within their respective module. In this regard, a cautious indication, still requiring more testing, could be that the items B10 (Quality of social responses) and A5 (Conversation) are possibly underestimated within M2 and might be considered to be additionally included in the cutoff calculation. The same is true for item B4 (shared enjoyment of interaction) within M4 (compare Figure 3).

On the other hand, there are items included in the ADOS/2 cutoff that ML rated as less relevant. For some items that only occur in one or two modules, relevance is likely underestimated in the ML models for statistical reasons. In contrast, it is evident that “D” items in particular (which would have the necessary power due to their cross-module occurrence) seem to be less relevant for the differentiation between ASD and Non-ASD, at least as listed within the RF results. Only D4 (“unusual repetitive interests or stereotyped behaviors”) appears relevant within the DT. If this item (in the drawn DT path sequence) is conspicuous, the calculated likelihood for ASD is 88.9% instead of 16.7% when the item is inconspicuous.

Importantly, features like age and IQ level also rank in the upper third of the most important features, suggesting a more direct influence on ASD diagnostic than it was apparent up to now. Although age is included in the ADOS/2 cutoff algorithm of M2, this focus is missing for the remaining modules. Moreover, IQ level is not taken into account in any of the four modules. In sum, especially with respect to the high amount of false positives (in case of individuals with below average IQ and ID) or else of false negatives (in case of individuals with above average IQ), we recommend clinicians to be aware of these potential confounding features in the diagnostic process. Based on the present data, it can be suggested that going through the short DT pathways, to reinsure the decision for or against ASD, may decrease the risk of false positives and false negatives in individuals with below and above average IQ levels remarkably. However, it is relevant to keep in mind that, in addition to or in interaction with IQ, further factors may influence the complex diagnostic process, including other differential or comorbid diagnoses (17, 18, 36, 45), female gender (46), parental psychiatric diagnoses (47), aspects of healthcare supply (48, 49), and experience of the diagnostician (50). Further, since IQ and language are observed to account for the heterogeneity in ASD and the variability in diagnostic and therapeutically outcomes, it appears relevant to consider also developmental trajectories of autistic symptom severity and adaptive functioning (51). For example, within analyses of the longitudinal EU-AIMS project, it was observed that higher age, lower IQ, and increased severity in (parent rated) social communication symptoms were predictive for lower adaptive functioning (52). Thus, the interplay between IQ, age, and symptom expression as well as their possible trajectories may be considered as well, if risks for false negatives or positives during ASD diagnostics were attempted to be reduced. However it was also concluded that individualized interventions need to focus on both aspects (symptom severity and adaptive functioning), since improvement in one domain does not ensure improvement in the other (51). In addition, even severely impaired children may improve substantially, so that they may enter adolescence with severity scores that are comparable to high functioning children. Prerequisite for this developmental trajectory is not to have an ID and to have a more educated, non-minority mother (53). In summary, the present and recent findings (7, 52) underline the high relevance of taking the IQ into account for the assessment of ASD symptomatology as well as for statements about course/prognosis and thus individual developmental trajectories and opportunities (54).

### Limitations

DTs are known to be very sensitive to changes in the training data: Minor modifications in the data provided for training can result in different trees. Therefore, in general, care has to be taken when drawing conclusions about the importance of attributes from DT. However, considering that the RF consists of DTs trained on randomly sub-sampled data, the impurity decrease measure can be regarded as a more robust measure of item importance. As the relevant features derived from the impurity decrease in the RF (Figure 3) are widely consistent with the relevant features obtained from the DT shown in Figure 2, the latter can be considered representative for similar data sets. As our analyses were applied in a cross-ADOS-module approach, further research is necessary to confirm the data as well as the drawn pathway within individual ADOS modules. A further limitation is that the ADOS/2 was part of the state-of-the-art diagnostic approach. Thus, the coincidence of ASD diagnosis and the ADOS/2 cutoff exceedance is possibly circular, confounding the results.

## Conclusions

In general, accuracies to predict BEC diagnosis of ASD yes vs. no between ADOS/2 cutoff and ML models are comparable. However, within sub-cohort analyses, i.e., individuals with below and above average IQ levels, the ADOS/2 algorithm was less accurate, resulting in 3 times higher risk for a false-positive ASD diagnosis in individuals with ID as well as 1.7 times higher risk for a false-negative ASD diagnosis in individuals with an above average IQ. This may be circumvented or decreased to the accuracy of individuals with average IQs by following the presented DT pathways, which could serve as a brief screening and as a solid decision-making basis for a subsequent all-encompassing diagnosis.

## Data Availability Statement

The original contributions presented in the study are included in the article/supplementary material, further inquiries can be directed to the corresponding author.

## Ethics Statement

The studies involving human participants were reviewed and approved by Ethics Committee of Department of Psychiatry, Campus Benjamin Franklin, Charité - Universitätsmedizin Berlin, Berlin, Germany (Az. 92/20). Written informed consent from the participants' legal guardian/next of kin was not required to participate in this study in accordance with the national legislation and the institutional requirements.

## Author Contributions

NW and ME executed the study idea, prepared and analyzed the data, wrote the first draft of the paper, and incorporated the comments and remarks from the co-authors. SS, IK-B, SR, and LP reviewed the paper, added comments, and rewrote parts of the paper. VR collaborated in all stages of the editing process of the final manuscript, added comments, and reviewed the paper from the first to the final draft. All authors contributed to the article and approved the submitted version.

## Funding

This work was funded by the German Federal Ministry of Education and Research (BMBF, grant number: FKZ 01EE1409B). Funding period: 2015–2021.

## Conflict of Interest

The authors declare that the research was conducted in the absence of any commercial or financial relationships that could be construed as a potential conflict of interest.

## Publisher's Note

All claims expressed in this article are solely those of the authors and do not necessarily represent those of their affiliated organizations, or those of the publisher, the editors and the reviewers. Any product that may be evaluated in this article, or claim that may be made by its manufacturer, is not guaranteed or endorsed by the publisher.
