# Corrigendum: Abilities and Disabilities—Applying Machine Learning to Disentangle the Role of Intelligence in Diagnosing Autism Spectrum Disorders

**DOI:** 10.3389/fpsyt.2022.908350

**Published:** 2022-04-25

**Authors:** Nicole Wolff, Matthias Eberlein, Sanna Stroth, Luise Poustka, Stefan Roepke, Inge Kamp-Becker, Veit Roessner

**Affiliations:** ^1^Department of Child and Adolescent Psychiatry and Psychotherapy, Faculty of Medicine, Technische Universität Dresden, Dresden, Germany; ^2^Institute of Circuits and Systems, Faculty of Electrical and Computer Engineering, Technische Universität Dresden, Dresden, Germany; ^3^Department of Child and Adolescent Psychiatry, Psychosomatics and Psychotherapy, Philipps University, Marburg, Germany; ^4^Department of Child and Adolescent Psychiatry and Psychotherapy, University Medical Center Göttingen, Göttingen, Germany; ^5^Department of Psychiatry, Campus Benjamin Franklin, Charité - Universitätsmedizin Berlin, Berlin, Germany

**Keywords:** autism spectrum disorders, IQ, intellectual disability, ADOS, machine learning, diagnostic, intelligence

In the published article (doi: 10.3389/fpsyt.2022.826043), there was an error in affiliations 1 and 2.

Instead of “Department of Child and Adolescent Psychiatry, Medical Faculty of the Technical University (TU) Dresden, Dresden, Germany” it should be “Department of Child and Adolescent Psychiatry and Psychotherapy, Faculty of Medicine, Technische Universität Dresden, Dresden, Germany.”

Instead of “Institute of Circuits and Systems, Faculty of Electrical and Computer Engineering Dresden, Technical University (TU) Dresden, Dresden, Germany” it should be “Institute of Circuits and Systems, Faculty of Electrical and Computer Engineering, Technische Universität Dresden, Dresden, Germany.”

The authors apologize for these errors and state that they do not change the scientific conclusions of the article in any way. The original article has been updated.

## Publisher's Note

All claims expressed in this article are solely those of the authors and do not necessarily represent those of their affiliated organizations, or those of the publisher, the editors and the reviewers. Any product that may be evaluated in this article, or claim that may be made by its manufacturer, is not guaranteed or endorsed by the publisher.

